# Berlin in Motion: Interprofessional teaching and learning for students in the fields of medicine, occupational therapy, physiotherapy and nursing (INTER-M-E-P-P)

**DOI:** 10.3205/zma001033

**Published:** 2016-04-29

**Authors:** Annerose Bohrer, Cornelia Heinze, Heidi Höppner, Ronja Behrend, Judith Czakert, Tanja Hitzblech, Ina Kaufmann, Asja Maaz, Jutta Räbiger, Harm Peters

**Affiliations:** 1 Evangelische Hochschule Berlin, Bachelor of Nursing, Berlin, Germany; 2 Alice Salomon Hochschule Berlin, course of studies for a B.Sc. in physiotherapy and occupational therapy, Berlin, Germany; 3 Charité - Universitätsmedizin Berlin, Dieter Scheffner Fachzentrum für medizinische Hochschullehre und evidenzbasierte Ausbildungsforschung, Berlin, Germany; 4 Supervisor, trainer of supervision, and coach (DGSv), mediator and systemic consultant of organization, Berlin, Germany; 5 Alice Salomon Hochschule Berlin, course of studies for a B.Sc. in phyisiotherapy and occupational therapy, Berlin, Germany

**Keywords:** Interprofessional learning, interprofessional education, cooperation, health professions

## Abstract

**Aim: **The Berlin project "Interprofessional teaching and learning in medicine, occupational therapy, physiotherapy and nursing" (INTER-M-E-P-P) pursues the goal of developing and testing interprofessional courses in an exemplary manner, and then implement these into their regular study programs.

**Method: **Under the direction of a steering committee of the participating institutions, professions and status groups, interprofessional courses were designed, carried out and evaluated. Specific to this project are the participation of students in the steering committee, and the accompanying of external supervision. The evaluation integrates the perspectives of all project participants, and combines quantitative and qualitative methods.

**Results: **INTER-M-E-P-P established cooperative structures between the participating universities and programs. Three courses were designed, taught and evaluated in an interprofessional manner. The various curricula, organizational patterns and locations of the study paths led to a great need for resources in regard to planning and implementation. This process can be made difficult by any stereotypes or preconceptions inherent to those doing the planning; however, under external supervision, the individual professional viewpoints can still be broadened and enriched.

**Conclusion: **A sustainable implementation of interprofessional education into the curricula of health science study programs is currently complicated by barriers such as different geographical locations and differing university regulations concerning study and testing. Implementation will require long-term support at the university as well as at political levels.

## Zusammenfassung

**Zielsetzung:** Das Berliner Projekt „Interprofessionelles Lehren und Lernen in Medizin, Ergotherapie, Physiotherapie und Pflege“ (INTER-M-E-P-P) verfolgt das Ziel, interprofessionelle Lehrveranstaltungen modellhaft zu entwickeln, zu erproben und in die Curricula der Studiengänge zu implementieren.

**Methodik: **Unter der Leitung einer institutions-, professions- und statusübergreifenden Lenkungsgruppe werden interprofessionelle Lehrveranstaltungen konzipiert, durchgeführt und evaluiert. Spezifisch sind die studentische Partizipation in der Lenkungsgruppe und die Begleitung durch externe Supervision. Die Evaluation integriert die Perspektiven aller am Projekt Beteiligten und kombiniert qualitative und quantitative Methoden.

**Ergebnisse: **INTER-M-E-P-P hat Strukturen zur Kooperation der beteiligten Hochschulen und Studiengänge aufgebaut. Drei Lehrveranstaltungen wurden interprofessionell konzipiert, durchgeführt und evaluiert. Die unterschiedliche curriculare, strukturelle und räumliche Verortung der Studiengänge führt zu einem hohen, ressourcenintensiven Planungs- und Umsetzungsaufwand. Stereotype und Rollenbilder der Planenden können den Prozess erschweren; unter externer Supervision jedoch die professionsspezifischen Blickwinkel erweitern und bereichern.

**Schlussfolgerung: **Eine nachhaltige Implementierung von IPL in die Curricula der Gesundheitsstudiengänge ist derzeit durch Barrieren wie die räumliche Trennung und verschiedene Studien- und Prüfungsordnungen erschwert. Sie benötigt dauerhafte Unterstützungsstrukturen auf hochschulischer und politischer Ebene.

## 1. Introduction

Interprofessional education (IPE) in health professions pursues the goal of enabling interprofessional collaboration (IPC) and contributing to a series of positive effects in the provision of health care: improvement of patients' care and safety, increasing the efficiency and effectiveness of medical teams, as well as promoting satisfaction among the members of interprofessional teams [1], [2]. Not least of all, IPE strives to positively influence student attitudes toward IPC and their relationships to other professions [1], [3], [2], [4]. 

In Germany, as regards practice and science, IPE has hitherto only been little developed. There are few study programs in the health professions in which multiple professional groups are taught with a focus on IPC [5]. For the most part, education in the fields of nursing, therapy, medicine and diagnostics takes place separately. However, the professionalization and academic training of nursing and therapeutic health sciences are lending IPE greater significance at the university level [5]. Clear demands are being voiced for more IPE: the advisory council’s expert opinion on cooperation and responsibility [6] stresses the importance of collaboration between the professions in the healthcare system. Likewise, the German Council of Science and Humanities supports IPE in selected joint courses and seminars [7], [8]. As an endowment foundation, the Robert Bosch Stiftung has set the course for increased collaboration by identifying 20 pathways, whereby cooperation among educational institutions is an essential means of attaining this [9]]. In a recent position paper of the relevant GMA committee, Walkenhorst et al. urgently call for interprofessional teaching strategies, along with the necessary curricular developments, structural changes, and focus on research and networking [5].

Seen internationally, IPE has long been the subject of increased attention and promotion, particularly following the 1988 WHO report, "Learning together to work together for health" [10]. At the turn of the twenty-first century, IPE was already widespread at Anglo-American universities [11] and as a result the body of research dealing with IPE has grown accordingly over the past 20 years. Focus has particularly been put on issues such as the extent to which IP course offerings displace profession-specific courses and lead to scheduling problems within the curriculum, whether or not student attitudes toward IPC are positively influenced over the long term through IPE, and which effects IPE has on outcome parameters, such as quality of care, patient safety, access to health care, and satisfaction among patients and healthcare workers [11], [12], [13], [14], [15]. Although the present state of research shows distinctly positive tendencies as a result of IPE, for instance in terms of job satisfaction [5], it also reveals conflicting observations and gaps in the existing research, among other things unanswered questions about which factors (personal background, time and location of post-secondary study, etc.) favourably influence IPE [2]. Overall, there is a need for more methodologically well-founded research on the topic of IPE [1], [2], [5].

In reference to sustainable integration of IPE into health care programs, published research identifies success factors that must be carefully considered when establishing IPE in Germany (see Table 1 (Tab. 1)). These success factors are based on the concept and implementation of this project in the light of our striving for a reference framework

## 2. Projekt description

The INTER-M-E-P-P (interprofessional teaching and learning in medicine, occupational therapy, physiotherapy and nursing) project is a joint developmental project between the Charité – Universititätsmedizin Berlin (Charité) – with its new modular curriculum of medicine, the Alice Salomon Hochschule Berlin (ASH) with its bachelor degree program in physiotherapy and occupational therapy, and the Evangelische Hochschule Berlin (EHB) with its bachelor degree program in nursing. The project is also part of the Robert Bosch Stiftung’s program entitled "Operation Team – Interprofessional Learning in Health Care Professions”, which ran from October 2013 through September 2015.

The aim of this project is to develop, implement, evaluate and anchor interprofessional course offerings on relevant topics into the curricula of the three participating degree programs. The project strives to give students the opportunity to become familiar with professional fields and profiles in joint courses, to become aware of and reflect on the roles expected of them and the other professions. In addition, students are given a chance to interact interprofessionally as part of the learning process. In terms of the persons, study programs and institutions involved in the development process, it is important to establish sustained cooperation and jointly meet the challenges of establishing IPE.

### 2.1. Project management and teamwork

**Figure 1:**
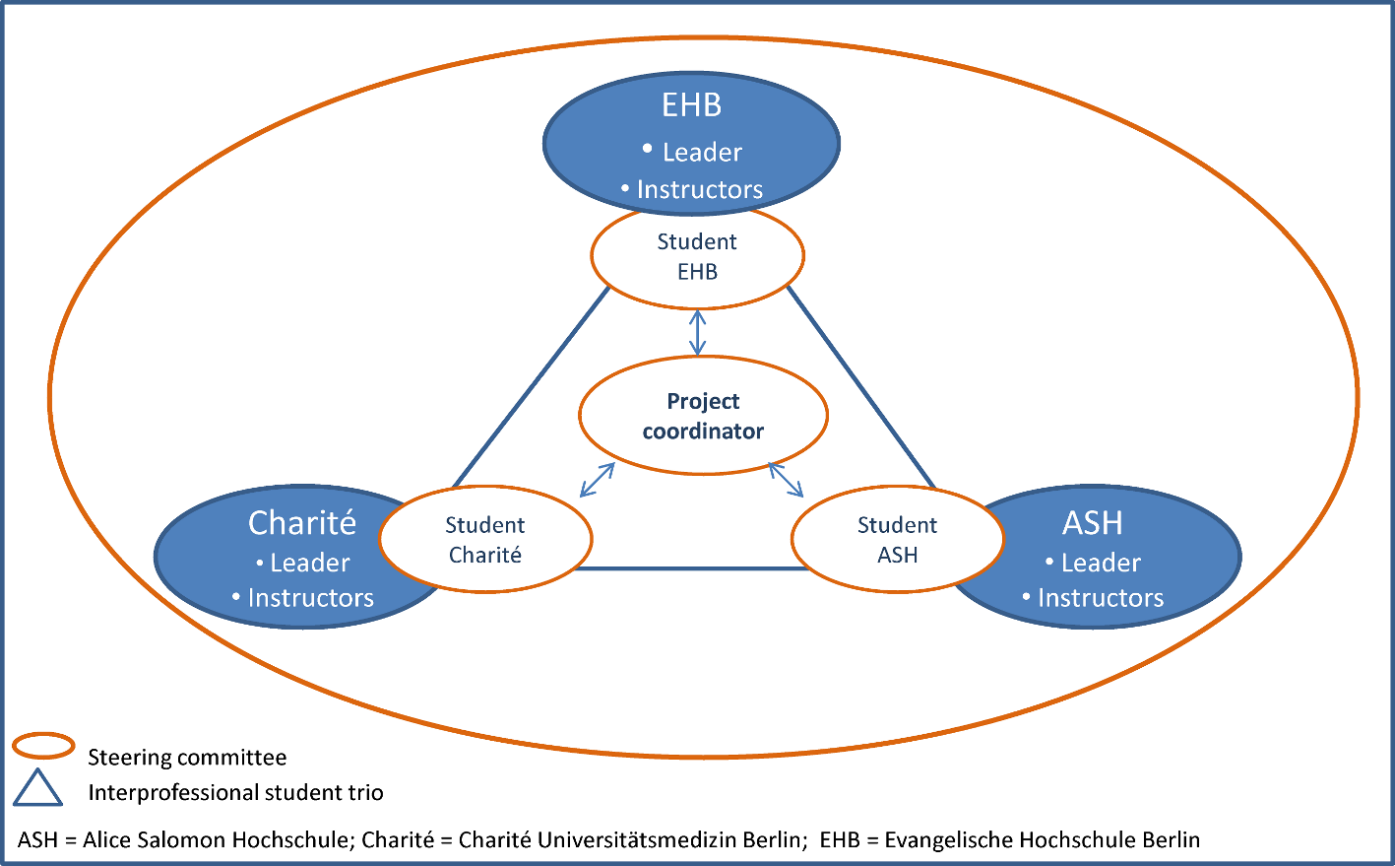
Project management and teamwork

A steering group consisting of representatives from medicine, occupational therapy, physiotherapy and nursing from each of the three institutions was formed as an essential component of the project. Project management was taken over by three professors from the three universities (see Figure 1 (Fig. 1)).

A central position was filled by a scientific and administrative project coordinator who coordinated the project. She was responsible for realizing the aims and decisions of the steering committee, putting courses and seminars into concrete form and working out the details, as well as scheduling and allotting classroom space for the course offerings. In the course of accomplishing this, the project coordinator was in constant contact with three student representatives who were involved over the entire process. The interprofessional student trio was not only part of the steering committee, but also a separate group which, in cooperation with the project coordinator, attended to the following tasks: collaboration in the design, conduction and evaluation of the course offerings, public relations, background research, developing input from the steering committee, and supplying ideas, discussion topics and feedback.

The planning and group work of the steering committee was accompanied over the entire project by a supervisor who provided support for team development from the very beginning. Along with classic supervision, elements of coaching, project management, process monitoring and advising, conflict moderation, and organizational consulting were also included. The challenges for all involved were above all of a logistical nature. They arose from the fact that three universities with different cultures, traditions, values and attitudes were working on a joint project over the very limited period of two years.

#### 2.2. Course design

Under the direction of the steering committee, three course offerings were designed. As starting point, relevant situations and the required competencies were identified, among them collaboration on managing hospital admission and discharge procedures, interprofessional case consultations, conducting patient rounds, and team conflicts. At the same time, study program curricula were examined for interdisciplinary topics which lent them to interprofessional education. In this case, the topics identified were those assessing the mobility and handling of patients with limited mobility. Existing courses were modified for the IP courses, and new teaching formats developed. The following courses were designed (see Table 2):

1. Principles for supporting patients with limited mobility
2. Team conflicts
3. Interprofessional collaboration in rehabilitation

Together with the instructors, teaching manuals were compiled which contained the course objectives and competencies, content and teaching methods. The instructors were given practical and theoretical support as needed regarding content and teaching.

As the courses underwent development, there were discussions about the various theoretical frameworks and teaching philosophies of each degree program. These include e.g. the understanding of competence or the principles of construction which belong to curricula. Shared principles for IPC were identified, such as a focus on pertinent and suitable examples of interprofessional situations in the work setting and the abilities to understand, cooperate and reflect. The question regarding a specific IPE teaching method for the participating health professions was raised by the project team, but still remains open.

Alongside the task of designing content, there was necessary – from the start of the project – communication about aims and backgrounds of IPE courses within the universities. This internal publicity served to increase the awareness and appreciation of upper-level university administrators, instructors, students and administrative employees, thus improving the conditions for implementing the new course offerings.

#### 2.3. Evaluation

Evaluation during the project took place on the one hand in terms of the planning and implementation processes, but also regarding the cooperation among those involved. Evaluation occurred in spoken form at regular intervals and every now and then in writing; a supervisor monitored the process. The experience and knowledge gained by the project group was formulated through structured reflection. The supervisor posed open questions as guidance and led methods for reflecting, such as “sitting on a chair” in which each group member individually gives feedback to three questions. The first question addressed positive experiences in working with the team and the communication in the INTER-M-E-P-P project during the previous year. The second question asked about ongoing work in regard to team cooperation and communication, while the third question focused on wishes and desires concerning teamwork and communication in the coming six months. The results were written down, analysed by the group, and agreements were reached about constructive changes.

On the other hand, the evaluation covered the newly designed course offerings themselves. These were analysed using qualitative and quantitative methods. At the beginning and end of the course which addressed the principles of supporting patients with limited mobility, the students filled out a survey with open-ended and closed questions. The responses to the open-ended questions were interpreted analytically.

A qualitative evaluation of the course dealing with team conflicts took place in which the students responded individually in writing, but also in their specific occupation groups to open-ended questions. In the same manner, the instructors participated in written evaluations, but for organizational reasons this was undertaken via email. Analysis of this qualitative data is still underway.

The lecture series on collaboration in patient rehabilitation was analysed informally in spoken form with the participating instructors and the steering committee.

## 3. Results

The major results are presented in the following sections to provide an overview of the successes and obstacles over the course of the entire project. Further publications will address the details of specific results, for instance those involving the three course offerings.

### 3.1. Implementation of the courses at the curricular and organizational level

A total of three interprofessional courses were offered to students of medicine, occupational therapy, physiotherapy, and nursing during the 2014/15 winter semester and the 2015 summer semester (see Table 2 (Tab. 2)).

The complexity of organizing the teaching demanded consistent adaptation of the IPE concepts to the structural aspects of the degree programs. Since course planning had to be done a year in advance, normal curricular integration of the IP courses was only partially possible, and implementation could not take place for all degree programs within the project timeline of two years (see Table 2 (Tab. 2)). The large cohorts of medical students (300 per semester versus 35 nursing students and 40 therapy students per year) also made it necessary to offer interprofessional and some mono-professional groups for the two courses “Supporting patients with limited mobility” and “team conflicts”. 

The efforts involved in planning and coordinating the interprofessional course offerings were very extensive. To perform the task of project coordination, a half-time position was required to cover the details involved in offering the courses, coordinating with instructors, scheduling and realizing the courses within the bounds of the times and spaces available. In particular, the liaison work between the three universities took up a great amount of time. Experience gathered from project implementation shows that clear contact partners and decision-makers at each institution are important for the incorporation of partly informal knowledge about the possibilities and limitations at the individual institutions.

Student employees were indispensable. At each of the three universities, a student employee was hired to spend 20 hours/month on the project for the full duration. Formally hiring the students ensured continuity of the employment relationship.

The interprofessional steering committee met regularly once or twice a month to work on planning, implementation and evaluation of the course offerings. A regularly scheduled, two-hour meeting on the second Friday of each month facilitated the actual meeting, and the professors from each degree program dedicated themselves over and above their normal teaching load. Results from the work process evaluations, conducted under the leadership of the supervisor, show that the supervision allowed challenges and conflicts within the steering committee to be addressed in a more open manner. Furthermore, it was possible for them to reflect on the fact that the logistical confines of the project (limits in terms of time, space, curricula) affected communication within the interprofessional working group.

#### 3.2. Course implementation and evaluation

In the following, exemplary results taken from two of the courses are outlined.

The 90-minute course on handling patients with limited mobility was team-taught in five interprofessional groups and taught by one instructor in 16 mono-professional groups consisting exclusively of medical students (see Table 2 (Tab. 2)). Overall, the course was taught by five different instructors having different academic backgrounds from the three universities. All of the students were taught according to the same teaching format in two large seminar rooms at the medical school. At the beginning of the course, the roles of the various professional groups were reflected. In a following group discussion, the students identified the specific goals of each profession during the provision of assistance and care to patients with limited mobility. After a short overview of the most important principles and the demonstration of various techniques for moving bed-ridden patients, the students had the opportunity to partner up and practice. The experiences from the practical exercises and the interprofessional exchange were reflected on at the end of the class session. At the end of the whole course, the students wrote short answers to the questions about what they particularly liked and disliked. All in all, 114 students from the interprofessional groups responded, and 112 from the mon-professional ones. In both groups, the chance to engage in practical exercises was rated positively. Both groups placed value on interprofessionalism as a topic, and profited from the knowledge they gained about the different responsibilities of each professional group. Only the interprofessional group had something to say about the experience of IPE. Some students in the mono-professional groups expressed criticism that there were no students from other health professions in their particular course. As a result, it was not possible to dispel prejudiced notions about other occupational groups. However, even in the interprofessional groups there was criticism to be heard that “stereotypes” were only mentioned, but no time could be made available to delve more deeply into the issue. The course schedule was criticized by both groups. Many participants in the interprofessional groups wished for more time to share experiences with students from other fields. Students of both groups criticized the lack of time for practical exercises related to patient mobility.

The interprofessional lecture series were held by interprofessional teams at each of the participating universities. Three lectures lasting 90 minutes each took place. In the first two lectures, an inadequate mix of students was apparent, and group discussions were rather halting. This was not only the result of frontal teaching in a large lecture hall, but also of varying levels of prior knowledge and expertise on the part of the students. During the final lecture, there was more success in achieving a better mix and more lively discussion among the students, for example by employing the targeted teaching strategies of scrambling the seating chart, having student introduce themselves, and using small groups in preparation for certain group discussions.

The lecturers in charge were generally satisfied with the course and the growth in knowledge among the students who attended. However, it was seen that most of the students who did attend were bound to do so as a result of curricular requirements. In order to achieve a more intense interprofessional exchange among students, an integration of the lecture series into the curriculum as a required (elective) course appears necessary.

Systematic analysis is still underway regarding the qualitative data gathered on the course covering team conflicts; however, it can already be stated that the participating students and instructors identify confronting stereotypes as a challenge. Pursuing more detailed information on this aspect does appear to be worthwhile.

The teaching and learning materials developed for the courses have been reviewed and revised on the basis of the evaluation results, and have been made available to the universities for further use.

## 4. Discussion

In this section, we critically discuss the project results in terms of success factors and challenges, as well as comment on the limitations of the project.

### 4.1. Success factors

The central success factors for the project are student participation and project management under external supervision.

#### Student participation

For Wilhelmsson et al. [16] the involvement of students belongs to a successful realization of IPE. Yet, in a review by Abu-Rish et al. [1] only 20% of the IPE studies evaluated included students.

Reasons for and benefits of including students in INTER-M-E-P-P can be summarized as:

1. A close connection with working practices,
2. An opportunity to introduce unconventional ideas within the group – free from institutional, status-related, or occupational limitations,
3. Knowledge of structural aspects of each degree program that can be used in planning and designing curricular content,
4. Augmentation of course evaluations to gather overall impressions about the attitudes and opinions of students before, during and after the courses, and
5. Close alignment with the needs of the student cohorts towards whom the course offerings are aimed.

Collaboration marked by respect represents a great opportunity for project success. At the same time, this requires a very conscious approach to communication, standards, rules and roles, namely matters that are facilitated by supervision.

##### Project management with external process monitoring (supervision)

Using supervision as a supportive measure was an important decision on the part of the project group. From the perspective of the supervisor and steering committee, this form of project management and collaboration in project teams (see 2.1) led not only to very intense and long discussions during the working phases, but also to better acceptance and knowledge of the perspectives and working habits of the other professions and degree programs. As a result of her critical thinking skills and solution-based approach, the supervisor was able to contribute to effective and constructive cooperation that focused on the resources available to all parties involved. She made it possible to identify the problems which arose over the course of collaborating, and then to process these on a meta-level. Due to the tight project schedule, even the supervisor was faced with limitations. More time for working through topics together such as the significance of stereotypes and role models within the project group would have been more ideal.

#### 4.2. Challenges

Major challenges for the project were seen in integrating IPE into the curriculum, shortages in staffing and time, as well as addressing role models and stereotypes.

##### Curricular integration of IPE

The curricular integration of IPE into all three degree programs – which was a primary aim from the beginning – could be only partially realized. The three programs differed in respect to the content, structure and scheduling of the modules for which IPE came into question. Coordination of lesson plans has been identified as a challenge in other studies [5].

In addition, the different student cohort sizes posed a challenge that could be met by forming interprofessional groups and mono-professional groups with medical students only.

According to Wilhelmsson et al., personal contact in small groups in a nonthreatening educational setting is crucial for a willingness to embrace IPE [16]. Five to ten participants is assumed to be an ideal group size [17], and something that could not be achieved in the lecture series as seen here. In contrast, the lectures offer the possibility to bring larger seminar groups together for IPE. In this case, personal contact among students should be promoted through targeted measures.

Scheduling belonged to the main difficulties faced in implementing IPE [1], i.e. the challenge of comparing the different course schedules of the student groups and finding times when not only the students but also the instructors were able to meet at a specific location. Alongside the challenge of creating time slots for IPE without much advance notice, topics were offered to accommodate the different academic levels so that in the IPE courses, students with varying prior knowledge and experience could come together. A possible solution to this could be a centrally coordinated, long-term curricular planning. It would appear sensible to determine specific spans of time for interprofessional course offerings. Resources are needed for organizational and administrative planning, as well as for scheduling and coordination. This undertaking was aggravated by the fact that the three different universities involved were located quite far away from each other (1 to 1.5 hours by car). Still, Buring et al. [18] are convinced that interprofessional cooperation between institutions in a comprehensive, even global manner can work successfully.

##### Staffing and time for IPE

The INTER-M-E-P-P project underscores the insight that making IPE a permanent feature at the participating universities will not happen on its own. Constructive project management was possible only as a result of third-party funding of a project coordinator position and three student employees and by the fact that the professors dedicated themselves above and beyond normal expectations.

Oandasan and Reeves [19] remark that participants (must) usually exercise their commitment to IPE in addition to their regular duties. To sustainably include interprofessional course offerings in degree program curricula, either as electives or requirements, this project has revealed that resources and administrative support at all three universities will be necessary over the long term, for instance to establish a central position for planning, coordination and designing IPE together with all stakeholders at the institutions. In addition, the work involved in IPE development and research must be counted toward the normal faculty workload to ensure that instructors are able to commit themselves over time to the work that needs to be done.

##### Addressing role models and stereotypes

One challenge remains: how do we approach stereotypes? Even instructors are socialized according to occupation, and they hold stereotypes more or less subconsciously. For this reason, it is important that planners and instructors address this as a team under the guidance of a supervisor.

During the course itself, enough time must be allotted to cover the topic of role models and to speak about stereotypes.

#### 4.3. Limitations

The primary aim of this project was to gather experience with interprofessional courses offered at three universities with three different degree programs and to lay the foundations for including IPE on a permanent basis. The project work took place in the form of action research: the stakeholders involved were not only the constructors of the interprofessional education and the cooperative network, but also the evaluators of their own work. This simultaneous carrying out of developmental work and evaluation led to enormous time pressure during the project, yet also allowed for systematic data analysis to take place afterwards. Some partial results during the project have already been incorporated into subsequent work, without the necessity to publish them as educational research.

## 5. Conclusion

The INTER-M-E-P-P project was able to identify important concrete possibilities but also limits of IPE for the participating institutions. Comprehensive project management by the project coordinator (central liaison between the participating institutions), constant student participation, and the collaboration on the interprofessional steering committee with external process monitoring (supervision) proved to be very productive.

The question about making the project a permanent part of the programs, and its recognition by the universities both remain central issues. In order to continue the work started here for an integration of IPE into health care curricula in a sustainable manner, it will be necessary to initiate supportive measures on both university and political levels, for instance in the form of agreements by the universities recognizing IPE as a legitimate field for research and development counting toward regular faculty duties. Together with university administrators at the participating institutions, a “Berlin manifest for cooperative learning in education and health care programs in Berlin” (*“Berliner Manifest für kooperatives Lernen in der Ausbildung und den Gesundheitsstudiengängen in Berlin”*) is currently being drafted, meaning that Berlin is in fact in motion and already off to a good start with the INTER-M-E-P-P project. Until consolidated policies at the university and state levels can be achieved, renewed third-party funding could buy more time to keep IPE alive in the meantime.

## Funding

The INTER-M-E-P-P project received a grant from the Robert Bosch Stiftung (project number 32.5.1316.0010.0).

## Competing interests

The authors declare that they have no competing interests.

## Erratum

The first name of the author Mrs. Kaufmann has been corrected.

## Figures and Tables

**Table 1:**
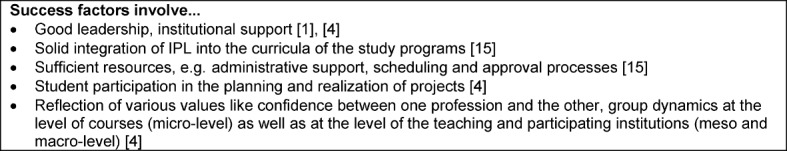
Success factors for implementing IPE

**Table 2:**
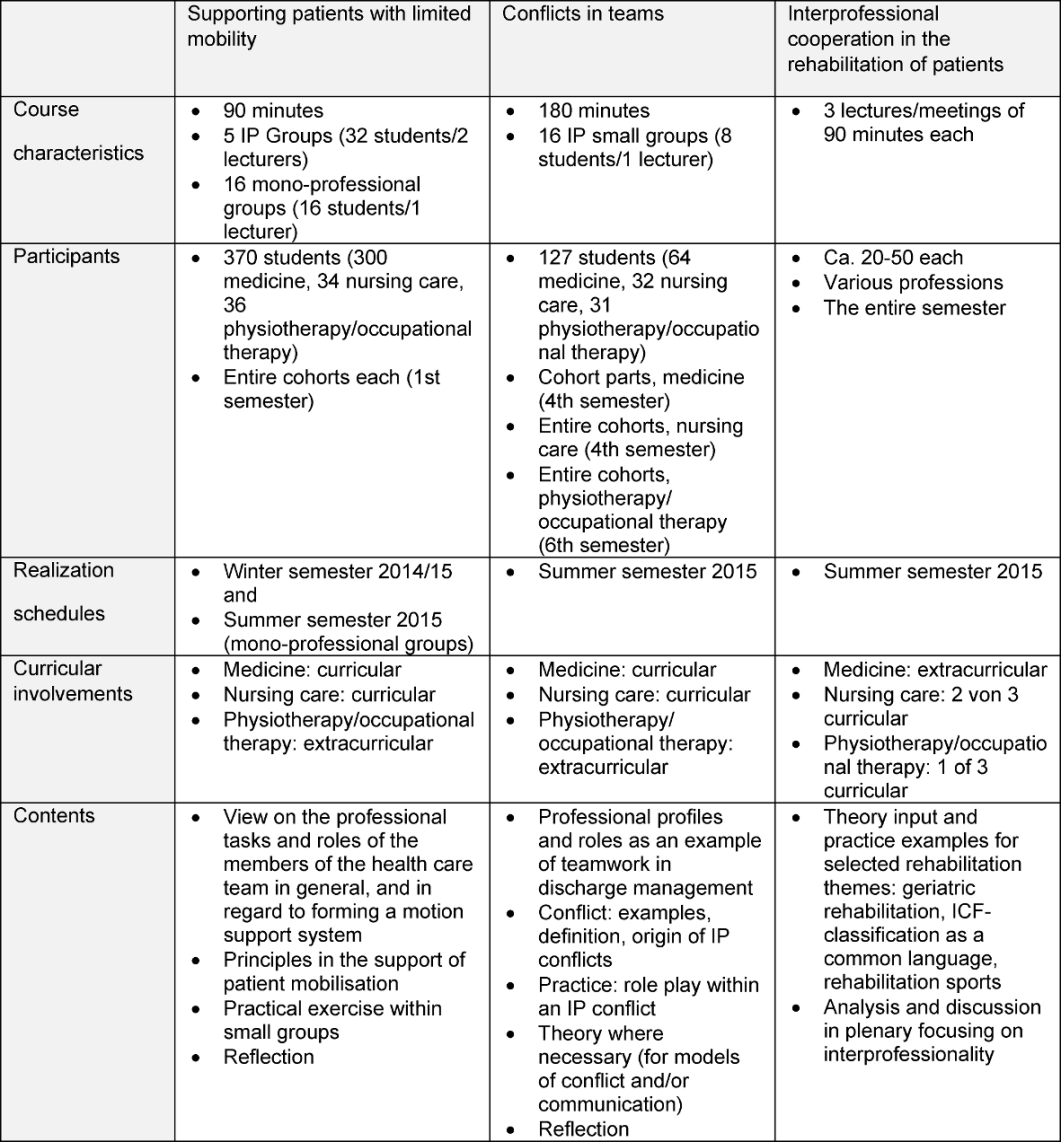
Overview of the interprofessional course offerings
